# Candidacidal Activity of Selected Ceragenins and Human Cathelicidin LL-37 in Experimental Settings Mimicking Infection Sites

**DOI:** 10.1371/journal.pone.0157242

**Published:** 2016-06-17

**Authors:** Bonita Durnaś, Urszula Wnorowska, Katarzyna Pogoda, Piotr Deptuła, Marzena Wątek, Ewelina Piktel, Stanisław Głuszek, Xiaobo Gu, Paul B. Savage, Katarzyna Niemirowicz, Robert Bucki

**Affiliations:** 1 Department of Physiology, Pathophysiology and Immunology of Infection, The Faculty of Health Sciences of the Jan Kochanowski University in Kielce, Kielce, Poland; 2 Holy Cross Oncology Center of Kielce, Artwińskiego 3, Kielce, Poland; 3 Department of Microbiological and Nanobiomedical Engineering, Medical University of Bialystok, Mickiewicza 2C, Bialystok, Poland; 4 Institute of Nuclear Physics, Polish Academy of Sciences, Radzikowskiego 152, Kraków, Poland; 5 The Faculty of Health Sciences of the Jan Kochanowski University in Kielce, Kielce, Poland; 6 Department of Chemistry and Biochemistry, Brigham Young University, Provo, Utah, United States of America; Louisiana State University, UNITED STATES

## Abstract

Fungal infections, especially those caused by antibiotic resistant pathogens, have become a serious public health problem due to the growing number of immunocompromised patients, including those subjected to anticancer treatment or suffering from HIV infection. In this study we assessed fungicidal activity of the ceragenins CSA-13, CSA-131 and CSA-192 against four fluconazole–resistant *Candida* strains. We found that ceragenins activity against planktonic *Candida* cells was higher than activity of human LL-37 peptide and synthetic cationic peptide omiganan. Compared to LL-37 peptide, ceragenins in the presence of DNase I demonstrated an increased ability to kill DNA-induced *Candida* biofilm. Microscopy studies show that treatment with LL-37 or ceragenins causes *Candida* cells to undergo extensive surface changes indicating surface membrane damage. This conclusion was substantiated by observation of rapid incorporation of FITC-labeled CSA-13, CSA-131 or LL-37 peptide into the more lipophilic environment of the *Candida* membrane. In addition to activity against *Candida spp*., ceragenins CSA-131 and CSA-192 display strong fungicidal activity against sixteen clinical isolates including *Cryptococcus neoformans* and *Aspergillus fumigatus*. These results indicate the potential of ceragenins for future development as new fungicidal agents.

## Introduction

Despite the variety of fungi that cause mycoses, *Candida* spp. remain the most common human fungal pathogens worldwide, causing both superficial and deep systemic invasions, including life-threatening bloodstream infections. *Candida albicans* followed by *C*. *glabrata*, *C*. *parapsilosis*, *C*. *tropicalis*, *C*. *krusei*, *C*. *lusitaniae*, and *C*. *guilliermondii* are the dominant species responsible for most forms of mycoses [1, 2]. *Candida* spp. are opportunistic microorganisms and are part of the normal human microbiota. They are present in the gastrointestinal tract, oral cavity and vagina, where they live as commensals but can cause infection in immunocompromised individuals [3]. Many factors such as indwelling central venous catheters, parenteral nutrition, chemotherapy, neutropenia, renal failure, hemodialysis, prolonged stay in the ICU, diabetes, and disruption of mucosal barriers predispose patients to mycosis [4]. Several virulence factors including adhesins, extracellular proteinase, the ability to make the morphological transition from blastospores to the hyphal form and biofilm formation have been investigated and linked to *Candida-*based infectiousness. Biofilm-forming capacity seems to play a crucial role in the pathogenesis of invasive candidosis (IC), especially candidemia connected with vascular catheters [5]. *Candida* strains form biofilms not only on indwelling medical devices but also on mucosal surfaces. Mucosal biofilms are mostly polymicrobial due to their formation from members of the endogenous human microbiota. Mature *Candida* biofilms consist of yeast cells and hyphal elements forming a three-dimensional network, adhered to the surface and embedded in a layer of extracellular matrix (ECM). From the medical standpoint the most important biofilm features are increased resistant to anti-microbial agents, protection from host defenses and long-term persistence [6–8]. *Candida* biofilm resistance is a multifactorial phenomenon, with various mechanisms acting together during the different stages of biofilm growth. The main element is the presence of ECM, which limits drug penetration. Others include: antibiotic inactivation by high metal ion concentration, low pH, phenotypic changes resulting from decreased growth and nutritional limitations, the presence within biofilms of metabolically inactive, non-dividing, dormant persister cells, up-regulation of different pathways associated with stress responses as well as mechanisms similar to conventional, planktonic antifungal resistance [9–11]. The *Candida* biofilm matrix consists primarily of extracellular polymeric substances including polysaccharides (ß-glucan) and extracellular DNA (eDNA) [12]. eDNA is an important matrix component not only of fungal but also bacterial biofilms that facilitates the adhesion to surfaces and binds with other biopolymers providing biofilm structural integrity and stability [13]. The induction of the morphological transition from yeast to the more invasive hyphal form is facilitated when eDNA is present [14]. Accordingly, recombinant deoxyribonuclease I (DNase I) decreases biofilm biomass [15].

Early diagnosis and appropriate antifungal treatment are essential for optimal management and successful outcomes in cases of *Candida* invasion, particularly those caused by antibiotic resistant strains. For systemic use, a choice of the polyenes, azoles, echinocandins and antimetabolites is available. However, due to the plethora of limitations associated with current antifungal treatments to cure mycosis, especially those caused by resistant fungi, new therapeutic strategies are right away needed.

Over the last years several novel approaches for treating fungal infections have emerged. Promising activity against pathogens was reported for antimicrobial peptides (AMPs) and their synthetic mimics. AMPs include a large number of multi-functional molecules present in many organisms including bacteria, fungi, plants, insects, worms and mammals. Due to their broad antimicrobial activity, amphiphilic character, rapid mode of action and low frequency in selecting resistant strains, they are interesting as potential therapeutic agents for topical and potentially systemic fungicidal applications [16]. Cathelicidin LL-37, the only cathelicidin found in humans, is an AMP produced by neutrophils, lymphocytes, macrophages and epithelial cells. It is released into body fluids in high concentrations during infection and inflammation. Apart from its antimicrobial activity, conditioned by membrane-permeabilizing ability, LL-37 also plays an important role in mucosal defense as the molecular component to the primary barrier against invasive pathogens [17]. LL-37 also participates in processes such as wound healing, tissue regeneration, angiogenesis, inhibition of neutrophil apoptosis and cytokine release [18]. Unfortunately, the use of LL-37 and other endogenous AMPs as potential drugs is limited by the high costs associated with large-scale synthesis, susceptibility to proteolysis by natural proteases, the potential to promote growth of some cancer cells and activation of autoimmune responses [19, 20]. Nevertheless, natural peptides serve as a pattern for the modification and development of novel, effective and cheaper therapeutics.

**Fig 1:**
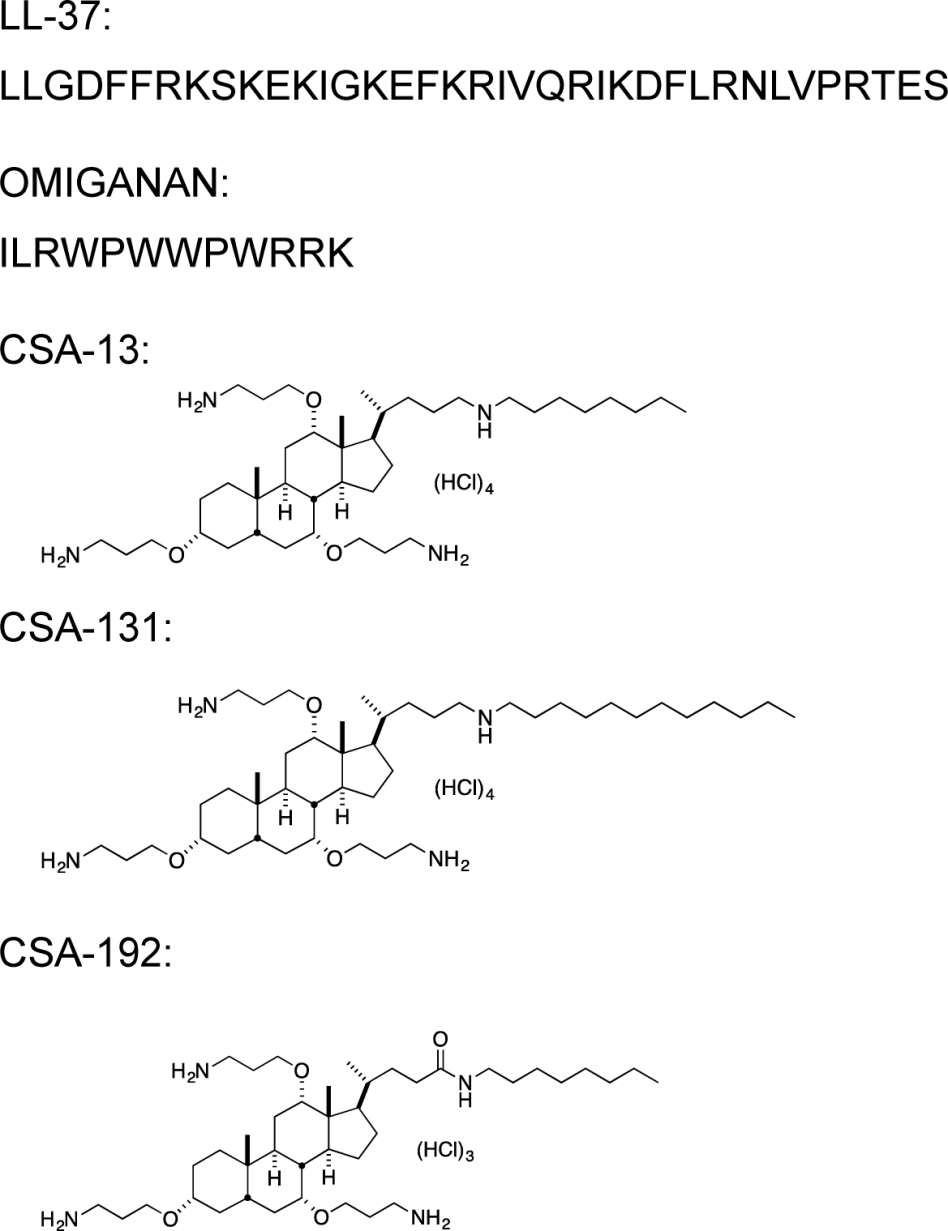
Structure of LL-37 peptide, omiganan and ceragenins CSA-13, CSA-131 and CSA-192. For amino acids, the one-letter code is used.

Among such new potential therapeutics with antimicrobial activity are ceragenins, amphiphilic derivatives of bile acids with covalently attached amines that mimic the amphipathic properties of endogenous AMPs. Ceragenins belong to cationic steroid antibiotics (CSAs) family. Due to their lipophilic nature they target pathogen membranes, causing morphological changes in membrane structure leading to cell death. Ceragenins show selective toxicity against microorganisms and are less expensive to prepare on a large scale than AMPs and are resistant to digestion by proteases [21–23]. Many ceragenins display broad-spectrum antibacterial activities against both Gram-positive and Gram-negative bacteria, including multi-resistant strains, parasites and some viruses [24–28]. The best known representative of ceragenin family is CSA-13; however, a variety of studies confirmed that other derivatives, including CSA-131 possess high antimicrobial activity, including activity against multidrug-resistant clinical isolates [29]. CSA-192 is a new member of ceragenin family, not yet tested in the context of fungicidal activity. The chemical structures of LL-37 peptide, omiganan and tested ceragenins, CSA-13, CSA-131 and CSA-192 are presented in Fig 1.

Strong antimicrobial activity has also been reported for synthetic antibacterial peptide omiganan pentahydrochloride (formerly known as MBI 226), which is an analogue of indolicidin [30]. Omiganan is active against over 1,400 clinical isolates of bacteria and 200 clinical isolates of yeast, including *C*. *albicans*, *C*. *glabrata*, *C*. *krusei*, *C*. *parapsilosis* and *C*. *tropicalis* [31]. Similarly to ceragenins and other AMP-based derivatives, the mechanism of omiganan action involves depolarization and disruption of cell membranes. Moreover, omiganan also acts via inhibition of protein, RNA and DNA synthesis, as observed in *Staphylococcus aureus* strains [31]. According to the database of U.S. National Institutes of Health, phase 3 of clinical trials on the efficiency of omiganan 1% gel in prevention of catheter-related bloodstream infections and topical skin antisepsis in healthy adult subjects have recently been completed [32].

In the present study, we aimed to assess the candidacidal activity of three ceragenins—CSA-13, CSA-131 and CSA-192 in comparison to LL-37 and omiganan against several strains of *Candida* in an experimental setting containing extracellular DNA (eDNA) as a factor promoting biofilm formation. We also assessed the potential of selected ceragenins to eradicate clinical and environmental isolates of pathogenic fungi.

## Materials and Methods

### Antifungal testing

Killing assays against *C*. *albicans* strains (CA 1407, CA 1408 and CA 1409, from the PAN, Wroclaw, Polish Collection of Microorganisms) and one clinical isolate of *Candida* spp. from patient diagnosed with oral mycosis were performed to compare the fungicidal activities of cathelicidin LL-37, omiganan (purchased from Polish Peptide Laboratory, Łódź and LipoPharm, Gdańsk, Poland), CSA-13, CSA-131 and CSA-192 (synthetized as described previously [33]). Briefly, fungal cells were grown to mid-log phase at 37°C, re-suspended in phosphate-buffered saline (PBS), and brought to 10^8^ CFU/mL. Cells were then added to PBS containing different concentrations of LL-37, omiganan, CSA-13, CSA-131 and CSA-192. After 1 h of incubation at 37°C the plates were transferred to ice and suspensions were diluted 10- to 1000-fold in PBS. Then, 10 μL aliquots were spotted on Sabouraud dextrose agar plates for overnight culture at 37°C and CFUs were determined. To assess the ability of the selected agents to kill fungal cells in different compartments of the human body, killing assays were performed in the presence human blood plasma, saliva, urine and pus (50% suspensions). In another set of experiments, changes of optical density during five hour incubation of *Candida* spp. in the presence of LL-37, CSA-13, and CSA-131 at dose of 10 μg/mL were measured as an additional method to assess cell viability and kinetic of growth. Yeast density was evaluated in OD_600_ = 0.1 using Labsystems Varioscan Flash (Thermo Scientific).

### Evaluation of MIC/MFC values

The microdilution method described in the guidelines of the Clinical Laboratory Standards Institute (CLSI) was used to determine minimum inhibitory concentrations (MICs) of LL-37, CSA-13, CSA-131, CSA-192, omiganan, amphotericin B and fluconazole [34]. Antifungal activity of the tested compounds against *Candida* strains was determined using pathogen cells in log-phase growth. Concentrations of the tested compounds ranged from 256 μg/mL to 0.5 μg/mL. MICs were determined visually as the lowest concentration of tested agents that showed no microbial growth after 24–48 h. In vitro fungicidal (MFCs) activities were determined by plating each sample on Sabouraud dextrose agar plates. To show the wide fungicidal activity of ceragenins towards a variety of fungi species, we performed another set of experiment where MICs of CSA-131 and CSA-192 against different yeasts (*Candida*, *Cryptococcus*, *Blastomyces*) and filamentous fungi (*Aspergillus*, *Scedosporium*, *Paecilomyces*, *Rhizopus*, *Apophysomyces;* clinical or environmental isolates) were determined. MICs values for all species apart from *Blastomyces* were evaluated by the microdilution method; for *Blastomyces* the macrodilution method was employed.

### Measurement of LL-37, CSA-13 and CSA-131 affinity to fungal membranes

To assess the affinity of cathelicidin LL-37 and ceragenins CSA-13 and CSA-131 to fungal membranes, compounds were labeled with fluorescein isothiocyanate (FITC) [35] and added to a suspension of *C*. *albicans* 1408 (OD_600_ = 0.1) to give a final concentration of 20 μg/mL [35]. The affinity of LL-37-FITC, CSA-13-FITC or CSA-131-FITC to cell membranes was assessed using fluorimetric measurements (Varioscan LUX Thermo Scientific) with excitation/emission wavelengths of 298/534 nm recorded during 6 min. The fluorescence register during 6 minutes was normalized to value obtained at the beginning of measured (0 min).

### Scanning Electron Microscopy (SEM)

*C*. *albicans* 1408 was resuspended in PBS to OD_600_ = 0.2. The cell suspension was incubated at 37°C for 60 min in varied concentrations of cathelicidin LL-37 (25, 50, 100 μg/mL) and ceragenin CSA-13 (10, 25 and 50 μg/mL). After incubation, the cells were centrifuged and washed 3 times at 3000g for 5 min with PBS. The resulting pellets were fixed in 0.5 mL of 2.5% glutaraldehyde in PBS at 4°C overnight. After incubation, the cells were washed twice with PBS and dehydrated through a graded ethanol series (50%, 70%, 90%, and 100%, 15 min in each) [36]. After lyophilization and gold coating (thickness approximately 20 nm), images were obtained using a scanning electron microscope (FEI Inspect S50).

### Atomic Force Microscopy (AFM)

*C*. *albicans* 1408 was resuspended in PBS (OD_600_ = 0.2), and incubated with LL-37 (25, 50 and 100 μg/mL) and CSA-13 (5, 10 and 25 μg/mL) at 37°C for 60 min. After incubation cells were centrifuged at 3000g for 5 min, washed in water, and centrifuged again. The pellet was resuspended in 20 μL of water and incubated on a mica surface precoated with 5% (3-Aminopropyl)triethoxysilane (APTES) in water until completely dry (ca. 30 min). AFM measurements were taken immediately. AFM images were collected using Nano Wizard 4 BioScience AFM (JPK Instruments, Germany) working in contact mode. ORC8 (Bruker) conical shaped tips with a nominal spring constant equal 0.38 N/m were employed. Initially, the tip was brought into contact with the surface of a *C*. *albicans* cell until a given deflection of the cantilever was reached. The scanning was then started with a constant velocity of 0.8 Hz. Three signals were recorded simultaneously while scanning the sample surface: topography, vertical deflection and lateral deflection of the cantilever, with the resolution of 256 pixels per line. Topography images serve as a qualitative assessment while vertical and lateral deflection uncover surface features with better clearness (data not shown).

### Activity against biofilm

*C*. *albicans* biofilms were grown for 24 and 48 h at 37°C with and without LL-37 (100 μg/mL), CSA-13 (5 μg/mL), CSA-131 (5 μg/mL) and DNA (0.2, 0.5, 1 mg/mL) and DNase I (Pulmozyme at 10 μg/mL). Each well was washed with 0.9% NaCl to remove planktonic cells. Biofilm mass was evaluated using the crystal violet (CV) staining (0.1%) method. The stain then was rinsed with deionized water and the plates were dried. Ethanol (90%, 100 μl) was added, and the optical density (OD) was measured at 570 nm [37].

### Statistical analysis

Collected data and differences were determined using the one-tailed Student’s t-test. Statistical analyses were performed using Statistica 10 (StatSoft Inc, Tulsa, OK, USA). P<0.05 was considered to be statistically significant. Results are the average from three to six measurements.

### Ethics statement

To perform evaluation of tested agents in the presence of body fluids, the materials were collected under IRB approval R-I-002/575/2013, Medical University of Bialystok. For both studies, all subjects provided written informed consent. All collected samples were anonymized.

## Results

### Ceragenins CSA-13, CSA-131, CSA-192, cathelicidin LL-37 and omiganan exert fungicidal activity against selected pathogenic fungi

**Fig 2:**
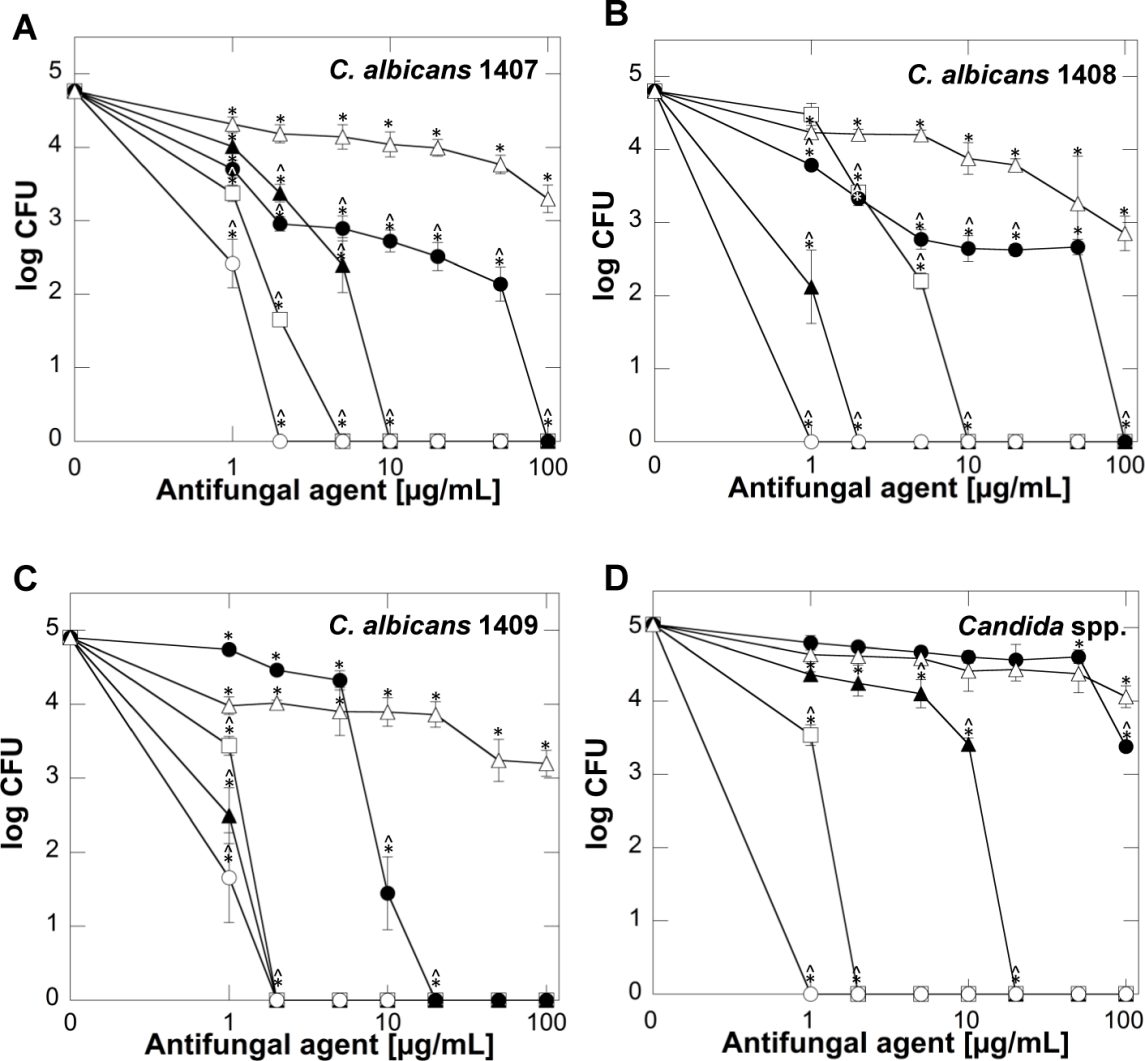
Candidacidal activity of cathelicidin LL-37 (filled circles), CSA-13 (empty squares), CSA-131 (empty circles), CSA-192 (filled triangles) and omiganan (empty triangles) against C. albicans 1407 (panel A), C. albicans 1408 (panel B), C. albicans 1409 (panel C) and Candida spp. (panel D). Error bars represent standard deviations from three to six measurements. * and ^ indicate statistical significance (*P*<0.05) compared to control (0 μg/mL of tested agents) and samples treated with omiganan, respectively. Statistical significance for the samples with log CFU = 0 was marked at the lowest dose of agent causing inhibition of growth of the fungus.

In the initial stage of the study we assessed the fungicidal activity of LL-37 and its non-peptide synthetic analogs, CSA-13, CSA-131 and CSA-192 against different *Candida* strains. Additionally, the fungicidal activity of tested agents, were compared to effect of omiganan. As shown in Fig 2, all of the tested ceragenins possess a much higher candidacidal activity than cathelicidin LL-37 and omiganan. It is noteworthy that fungicidal activity of the compounds is dependent on the *Candida* strain. The strongest biological activity was observed against the *C*. *albicans* 1409 strain; fungus growth inhibition was recorded at 2 μg/mL of CSA-13, CSA-131 and CSA-192 and 20 μg/mL of LL-37 peptide. Notably, a dose of omiganan at 100 μg/mL was insufficient to inhibit fungal growth (Fig 2C). Importantly, CSA-13 displays high candidacidal activity against all tested fungal strains in contrast to LL-37 peptide, whose activity was significantly lower for *C*. *albicans* 1407, *C*. *albicans* 1408 and *Candida* spp. strains (Fig 2A, 2B and 2D). Additionally, ceragenins MIC/MFC values were significantly lower, when compared to fluconazole (confirming strains resistance) and were comparable to amphotericin B, which indicates strong fungicidal activity of these agents (Table 1).

**Table 1:** Minimal inhibitory concentration (MIC, μg/mL) and minimal fungicidal concentration (MFC μg/mL) of tested agents, against tested *Candida* strains.

Strain	LL-37 MIC/MFC μg/mL (μM)	CSA-13 MIC/MFC μg/mL (μM)	CSA-131 MIC/MFC μg/mL (μM)	CSA-192 MIC/MFC μg/mL (μM)	Omiganan MIC/MFC μg/mL (μM)	Amphotericin B MIC/MFC μg/mL (μM)	Fluconazole MIC/MFC μg/mL (μM)
*C*. *albicans* 1407	64/128 (14.2/28.5)	0.5/1 (0.6/1.2)	8/16 (9.1/18.2)	2/4 (2.4/4.9)	64/128 (36.0/72.0)	1/1 (1.1/1.1)	128/128 (418.0/418.0)
*C*. *albicans* 1408	>256/>256 (>57.0)	1/1 (1.2/1.2)	8/8 (9.1/9.1)	1/2 (1.2/2.4)	64/128 (36.0/72.0)	1/1 (1.1/1.1)	16/32 (52.2/104.5)
*C*. *albicans* 1409	>256/>256 (>57.0)	4/4 (4.9/4.9)	32/32 (36.5/36.5)	2/4 (2.4/4.9)	64/128 (36.0/72.0)	0.5/1 (0.5/1.1)	16/32 (52.2/104.5)
*Candida* spp.	64/128 (14.2/28.5)	0.5/1 (0.6/1.2)	8/16 (9.1/18.2)	1/2 (1.2/2.4)	128/128 (72.0/72.0)	1/2 (1.1/2.2)	>256/>256 (>835.9/>835.9)

**Fig 3:**
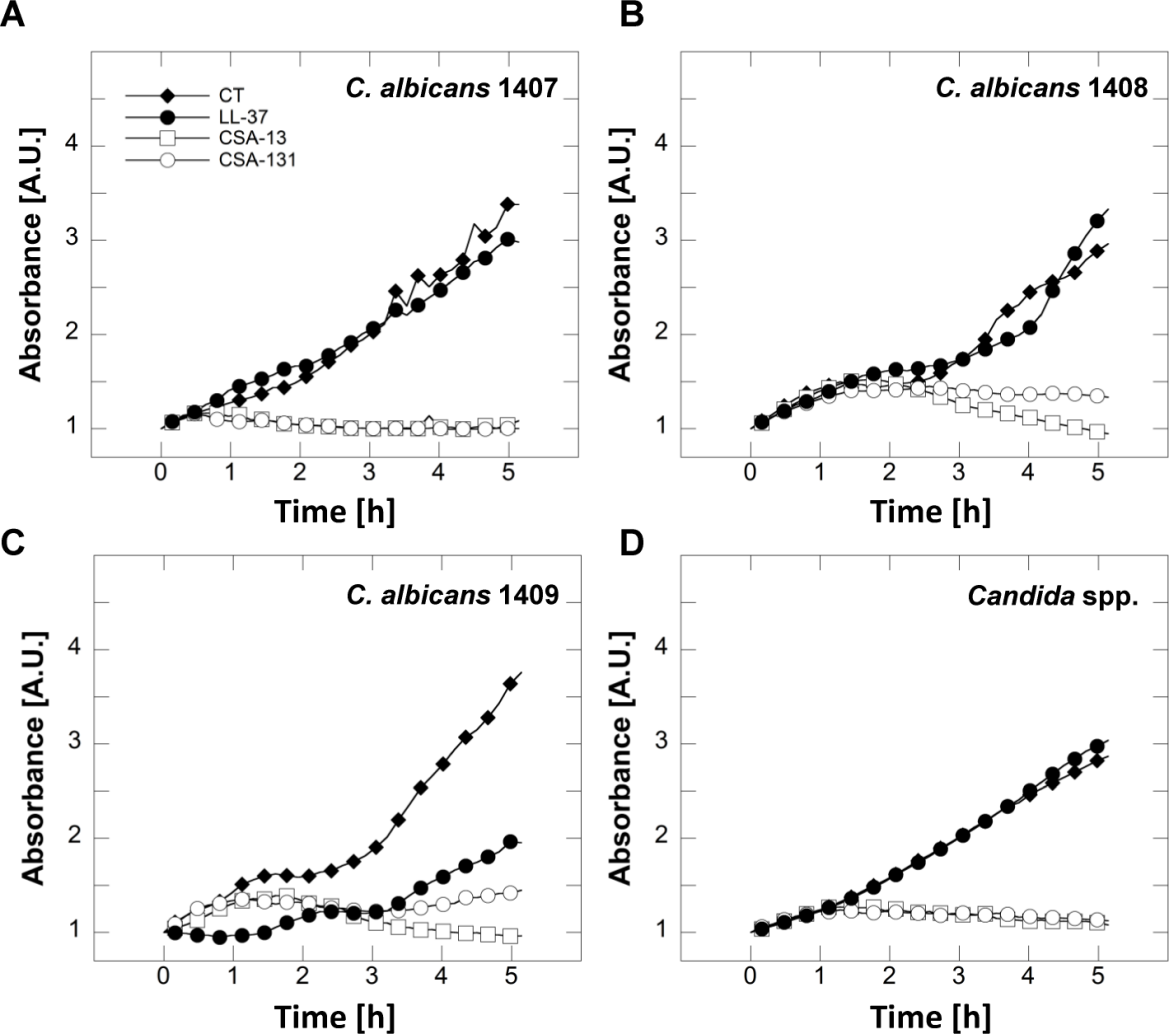
Growth curve of *C*. *albicans* 1407 (panel A), *C*. *albicans* 1408 (panel B), *C*. *albicans* 1409 (panel C) and *Candida* spp. (panel D) indicated by filled diamonds (control) and growth in presence of LL-37 (filled circles), CSA-13 (empty squares) and CSA-131 (empty circles). The absorbance (OD_600_) was monitored during five hours. Representative results from one of four experiments are shown.

To confirm these results, *C*. *albicans* cell suspensions were incubated in the presence of LL-37 and ceragenins and the level of fungal growth was determined using a spectrophotometric method (Fig 3). When compared to non-treated controls, LL-37 did not significantly affect fungal growth over five hours of incubation. In contrast, CSA-13 and CSA-131 strongly inhibited fungal cell proliferation for all tested strains.

**Fig 4:**
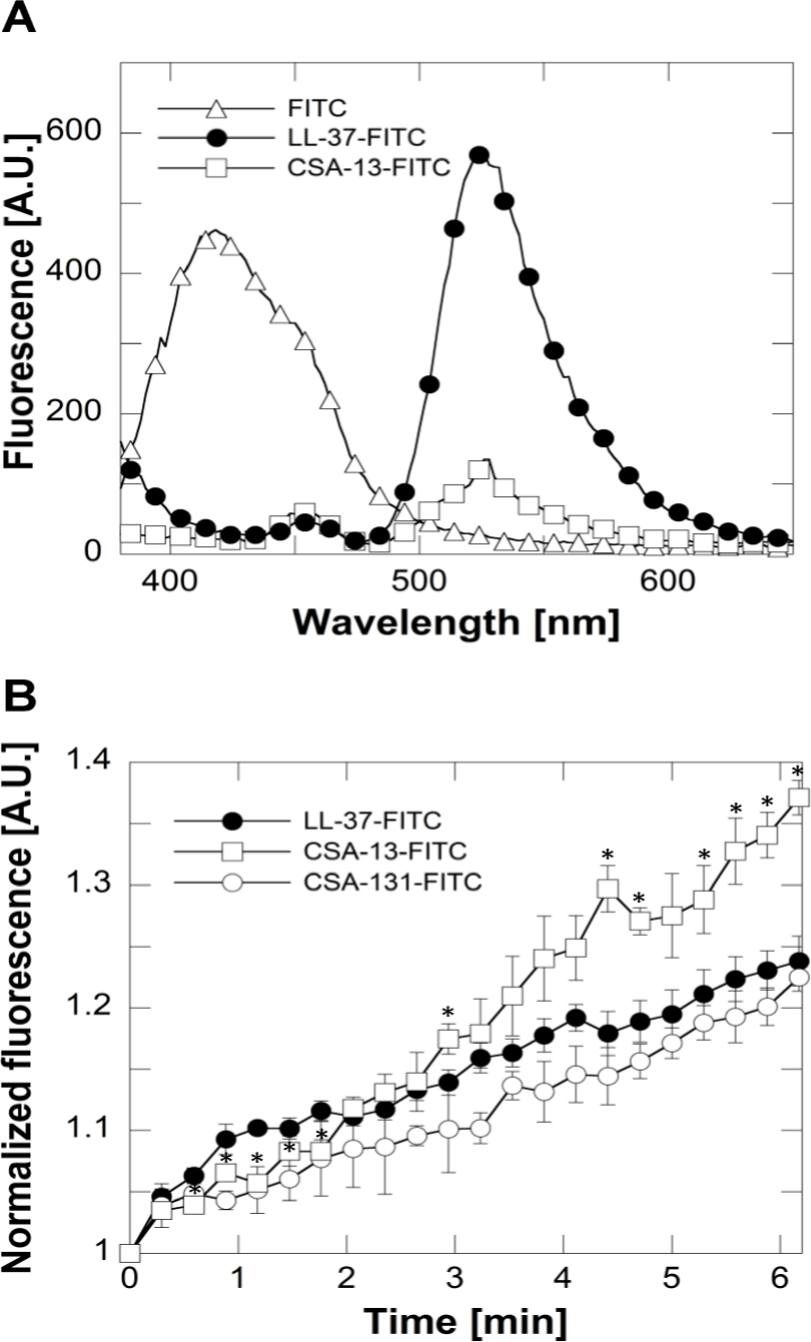
Fluorescence emission spectra of FITC (empty triangles), FITC-labeled LL-37 (filled circles) and FITC-labeled CSA-13 (empty squares) (panel A). Affinity of FITC-labeled agents to fungal membranes were monitored by recording the changes of fluorescence over 6 min. Error bars represent standard deviations from three measurements (panel B). * indicates statistical significance (*P*<0.05) compared to affinity of LL-37 to fungal membrane.

To assess how the killing properties of LL-37 and ceragenins correlated with the affinity of these compounds for fungal membranes, an affinity assay using FITC-labeled agents (LL-37 and ceragenins: CSA-13 and CSA-131) was performed. To confirm the functionalization of the compounds by FITC, labeled agents were analyzed by fluorescence spectroscopy. The emission spectra of FITC-labeled LL-37 peptide and CSA-13 were recorded using Spark® 20M microplate reader (Tecan Group Ltd., Switzerland) and are presented on Fig 4A. Fig 4B displays, LL-37 and its mimics (CSA-13 or CSA-131) affinity towards *C*. *albicans* 1408 membrane. The greater candidacidal activity of CSA-13, when compared to LL-37, correlated with the higher affinity of this compound for the fungal membrane.

**Fig 5:**
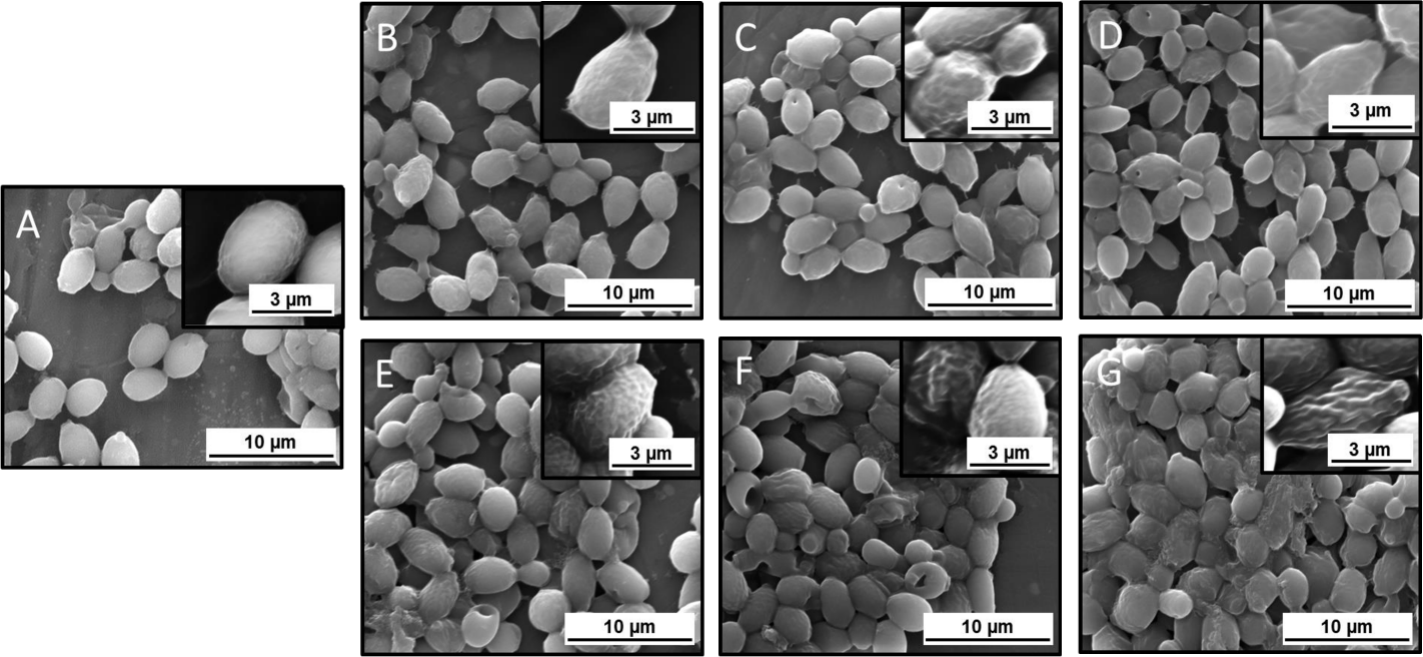
Scanning electron microscope (panels A-G) photomicrograph of the untreated *C*. *albicans* 1408 (panel A) and after addition of cathelicidin LL-37 (at 25 μg/mL panel B, 50 μg/mL panel C and 100 μg/mL panel D) or ceragenin CSA-13 (10 μg/mL panel E, 25 μg/mL panel F and 50 μg/mL panel G).

**Fig 6:**
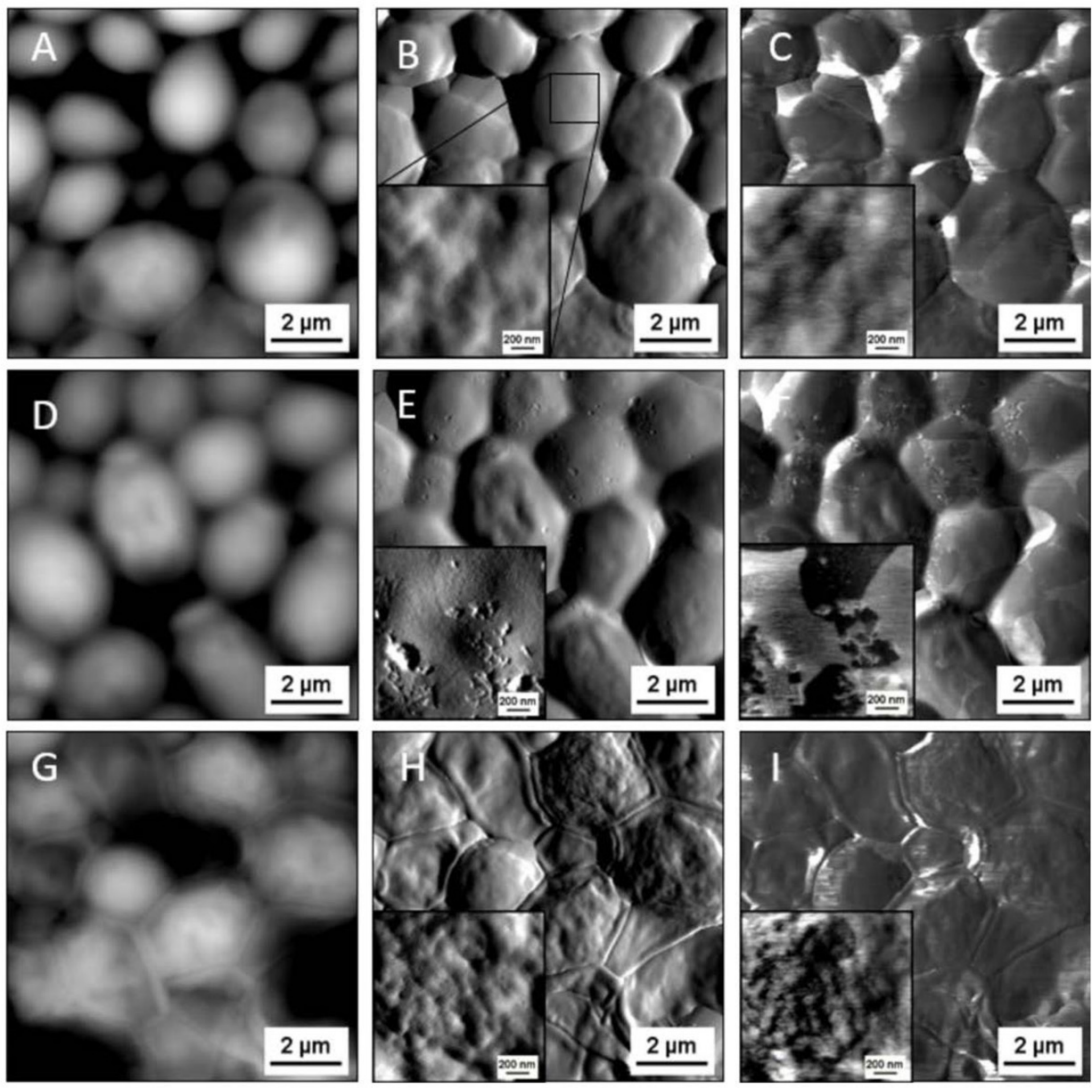
Atomic force microscopy measurements of untreated (A-C), treated with 100 μg/mL LL-37 (D-F) and 25 μg/mL CSA-13 (G-I) *Candida albicans* 1408 cells. Panels A, D, G present topography images, panels B, E, H vertical cantilever deflection and panels C, F, I lateral cantilever deflection. Small images inside display local changes in surface morphology of a single cell (scale bar 200 nm). Representative results from one experiment are shown.

To visualize the effects of the tested compounds on the vegetative form of *C*. *albicans* 1408, scanning electron microscopy (SEM) and atomic force microscopy (AFM) were used (Figs 5 and 6). Compared to untreated controls, SEM and AFM images of fungi treated with varied concentrations of LL-37 and CSA-13 showed morphological alternations. Fungal cells treated with LL-37 (Fig 5B–5D) and CSA-13 (Fig 5E–5G) were characterized by more wrinkled cell surfaces. Some parts of the treated cells also changed shape, changing from oval to more elongated forms. Importantly, changes in cell morphology were dose-dependent. Furthermore, CSA-13 altered the structure of the cell membrane to a greater extent and at lower doses than the peptide. AFM evaluation revealed additional aspects of morphological changes and significant morphological differences between the *C*. *albicans* cells untreated and treated with LL-37 and CSA-13. LL-37-treated cells exhibit small, crack-like break in the cell surface (Fig 6D–6F) whereas the CSA-13 treated cells show increased surface wrinkling (Fig 6G–6I). This effect can be observed mostly on vertical deflection images (Fig 6B, 6E and 6H). Lateral deflection images show a lateral bending of the cantilever when scanning across the sample. These images can be related to friction forces acting between the sample and cantilever tip. The differences in lateral deflection images for LL-37-treated cells (Fig 6F) allows to distinguish domains with a different friction. This effect is not observed for CSA-13 treated cells (Fig 6I), where lateral deflection images correspond to topography and vertical deflection images. This may indicate two different mechanisms for antimicrobial activity of LL-37 and CSA-13. Since *Candida* cells for SEM imaging were fixed after the treatment, whereas cells for AFM imaging were air dried without fixation, it should be noted that differences between SEM and AFM outcomes could result from dissimilarities in preparation steps. Additionally, we suggest that alternations in morphological features observed for LL-37 below the MIC/MBC values obtained in the previous stages of our research (>256μg/mL), results from preparation of samples to AFM studies in water, which could promote the antimicrobial effect of this peptide due to osmotic effects [38]. Importantly, morphological changes showed by our results are consistent with previous reports indicating alternations in morphological structure of fungal cells after treatment with fungicidal agents [39].

As indicated by data included in the Table 2, ceragenins CSA-131 and CSA-192 possess high potential to eradicate broad spectrum of yeast and filamentous fungi.

**Table 2:** Minimal inhibitory concentration (MIC, μg/mL) of CSA-131 and CSA-192 against clinical and environmental isolates of yeast or filamentous fungi.

Clinical isolate	CSA-131 MIC [μg/mL]	CSA-192 MIC [μg/mL]
*Candida parapsilosis*	2	2
*Candida krusei*	2	1
*Paecilomyces variotii*	2	2
*Cryptococcus neoformans*	1	1
*Cryptococcus neoformans*	1	1
*Cryptococcus neoformans*	1	1
*Aspergillus fumigatus*	4	16
*Aspergillus fumigatus*	4	16
*Aspergillus fumigatus*	4	16
*Scedosporium apiospermum*	4	16
*Scedosporium apiospermum*	2	2
*Rhizopus arrhizus*	4	8
*Rhizopus arrhizus*	2	8
*Rhizopus arrhizus*	2	8
*Blastomyces dermatitidis*	2	4
*Blastomyces dermatitidis*	1	4
*Blastomyces dermatitidis*	1	1
*Apophysomyces*	4	2

### CSA-13 and CSA-131 inhibit DNA-mediated growth of biofilm

**Fig 7:**
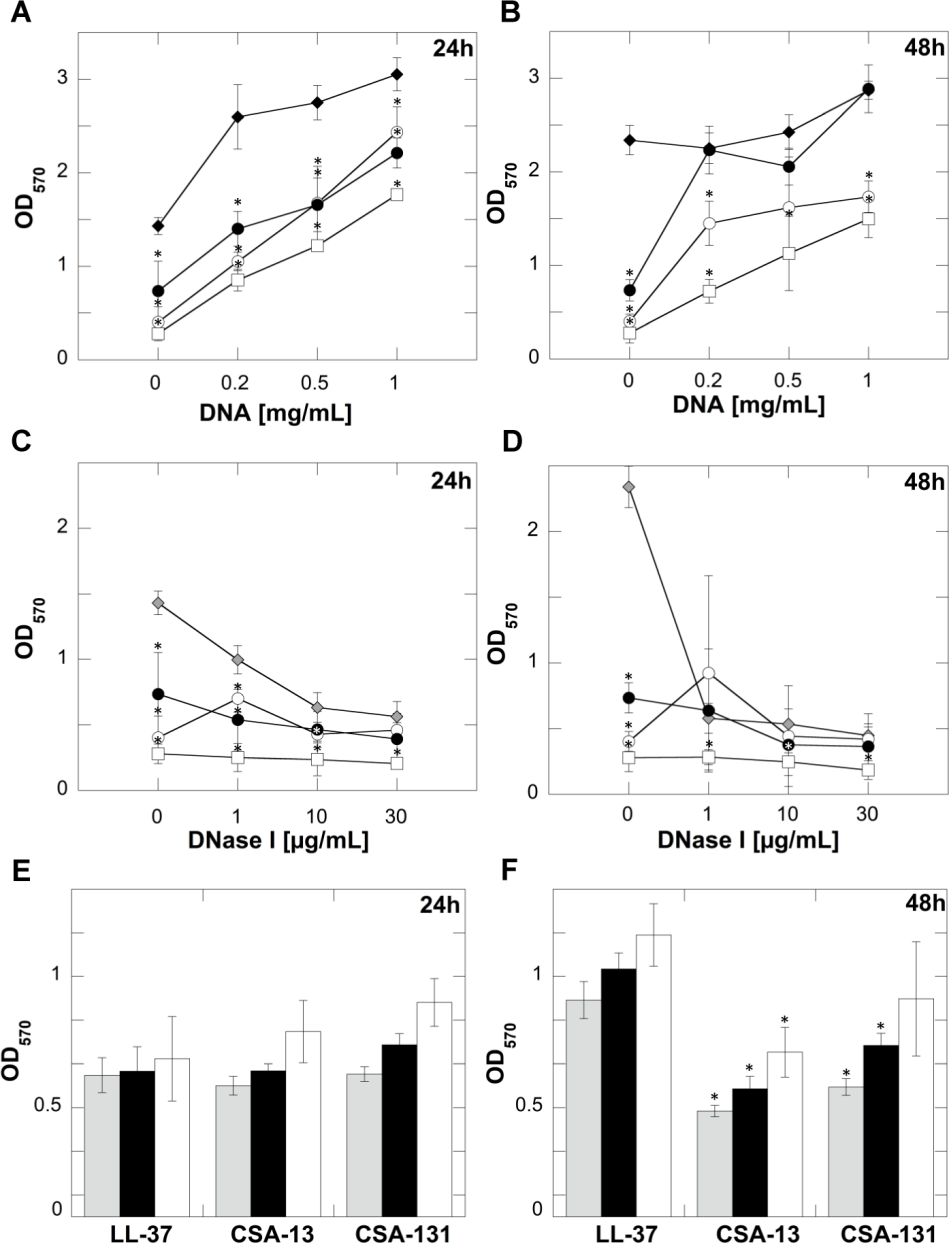
*C*. *albicans* biofilm formation in the presence of DNA (filled diamonds) or DNA and LL-37 (filled circles), CSA-13 (empty squares) or CSA-131 (empty circles) after 24 h (panel A) or 48 h of growth (panel B). Inhibitory effect of DNase I (grey diamonds) or its combination with LL-37 (filled circles), CSA-13 (empty squares) or CSA-131 (empty circles) on *C*. *albicans* biofilm formation after 24 h (panel C) and 48 h of growth (panel D). *C*. *albicans* biofilm formation at 24 h (panel E) and 48 h (panel F) with combination of DNA 0.2 mg/mL and DNase I 10 μg/mL (grey columns), DNA 0.5 mg/mL and DNase I 10 μg/mL (black columns) or DNA 1 mg/mL and DNase I 10 μg/mL (white columns) in the presence of LL-37 (100 μg/mL), CSA-13 (5 μg/mL) and CSA-131 (5 μg/mL) respectively. Each result corresponds to the average of three different isolates of *C*. *albicans* (1407, 1408 and 1409). Error bars represent standard deviations from three to six measurements. * indicate statistical significance (*P*<0.05) compared to control (samples with 0 μg/mL of tested agents) (panels A-D) or compared to samples treated with LL-37 (panels E and F).

To measure the impact of polyelectrolytes on the fungicidal activity of LL-37 and ceragenins, we performed an assay in which fungal biofilms were formed in the presence of DNA and the tested agents. According to results shown in Fig 7A and 7B, ceragenins CSA-13 and CSA-131 significantly inhibited the formation of both young and mature biofilms even in the presence of high DNA concentrations (1 mg/mL). The relative biofilm masses formed in the presence of both ceragenins was nearly 2-fold lower when compared to non-treated control. LL-37 did not maintain its inhibitory effect and biofilm mass formed after 48 h of incubation was comparable to control. Previous studies suggest that DNase I may be used as a therapeutic compound for the treatment of biofilms formed by bacteria and fungal pathogens [15, 40]. Accordingly, CSA-13 displays strong synergism with DNase I against *C*. *albicans* biofilms. As expected, incubation of samples with DNase I and ceragenins inhibited biofilm formation to a greater level than with DNase I alone. Results shown in Fig 7C and 7D, suggest that CSA-13 has the greatest potential as a combination treatment with DNase I, since the relative biofilm mass after incubation with this compound was the lowest. Moreover, this effect was dose-dependent, since doses ranging from 1 to 30 μg/mL led to a decrease in biofilm formation to nearly non-detectable levels, suggesting that even low doses of CSA-13 are effective in inhibiting biofilm growth. The increased anti-biofilm activity of DNase I/ceragenin combination was observed for mature biofilms as well; however, for biofilms formed over 48 h, the reduction of biofilm mass was not strongly pronounced. Anti-biofilm activity of the tested compounds was also observed for samples treated with LL-37, CSA-13 or CSA-131 and incubated with a mixture of various concentrations of DNA and DNase I at a concentration of 10 μg/mL (Fig 7E and 7F). It was confirmed that increased DNA concentrations did not alter the biological activity of these compounds. These results suggest that CSA-13 and CSA-131 possess high potential for treatment of mature fungal biofilms.

### The candidacidal activity of LL-37, CSA-13 and CSA-131 is altered in different body fluids

**Fig 8:**
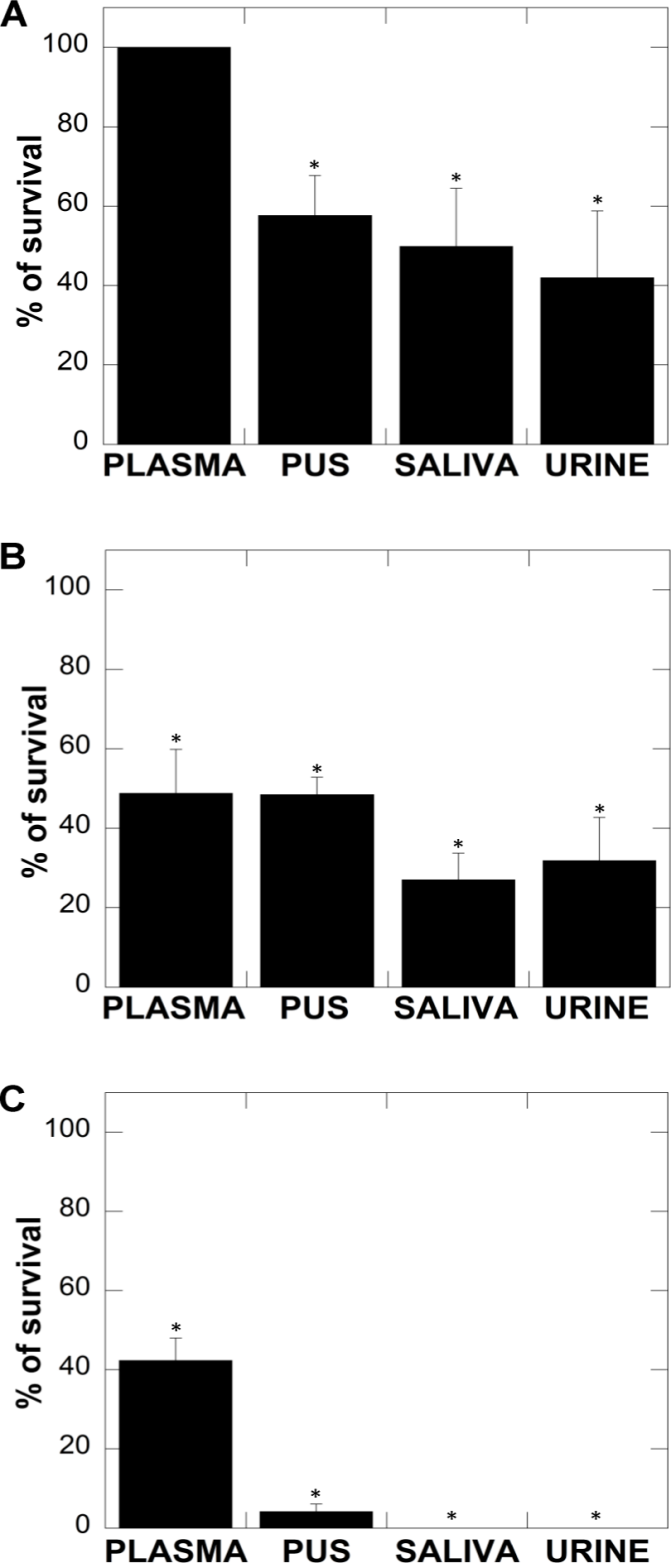
Antifungal activity of LL-37 (panel A), CSA-13 (panel B) and CSA-131 (panel C) against *C*. *albicans* in human body fluids (50% of plasma, pus, saliva or urine). Black columns indicate the percent of survival of fungal cells after treatment with 100 μg/mL of LL-37 peptide (panel A), 5 μg/mL CSA-13 (panel B) and 5 μg/mL CSA-131 (panel C) in the presence of body fluids. Each value corresponds to the average of results with three different isolates of *C*. *albicans* (1407, 1408 and 1409). Error bars represent standard deviations from four to six measurements. * indicate statistical significance (*P*<0.05) compared to untreated samples (0 μg/mL of tested agents).

An important criterion for the effective treatment of fungal infections is maintenance of satisfactory activity of antifungal agents in experimental settings mimicking infections sites. Considering the studies reporting the inhibitory effect of a variety of factors present at infection sites on the antimicrobial activities of LL-37 and its non-peptide synthetic analogs, incubation of fungal suspensions with different body fluids supplemented with tested agents was performed to determine if these agents would maintain activity in complex matrices. As shown in Fig 8, the fungicidal properties of LL-37 and both CSA-13 and CSA-131 ceragenins differ in human body fluid. Data presented in Fig 8A indicate the candidacidal activity of LL-37 is entirely compromised in blood plasma. Moreover, its biological activity is significantly decreased in pus, saliva and urine, which potentially limits the employment of LL-37 in clinical applications. The fungicidal activities of CSA-13 and CSA-131 were less affected by blood plasma, since they twice reduced the number of CFUs, when compared to untreated controls as well as to cathelicidin LL-37. The survival rate ranged from 47% to 30% for strains incubated in pus, saliva and urine following the addition of CSA-13 suggesting that higher doses of these agents may be sufficient to effectively eradicate *Candida* infections, even in clinical settings (Fig 8B). Most importantly, the compounds present in pus, saliva and urine did not alter the candidacidal activity of CSA-131, which suggest that this compound may be useful in the clinical treatment of fungal infections (Fig 8C).

## Discussion

Due to the significant increase in resistance among many species of clinically important microorganisms, there is a need for the development of more effective and safe antimicrobial drugs. Reduced susceptibility to antifungals (mainly fluconazole and echinocandins) associated with their growing use in prophylaxis and treatment has become a challenging issue in the management of fungal infections [41]. AMPs, both natural and synthetic, as well as non-peptide synthetic compounds mimicking the amphiphilic morphology of antibacterial peptides, such as ceragenins, offer a promising platform for developing new fungicidal agents. Several studies have shown that human cathelicidin LL-37 and ceragenins are effective against different pathogens, particularly bacteria, including multidrug-resistant strains [42–44]. In contrast to the effects on bacteria, the antifungal activity of LL-37 and other human AMPs is less well defined. Only a few studies indicate their effectiveness against fungi [45, 46]. Promising results have been achieved using omiganan, an AMPs derivative of indolicidin, a compound that displays membrane-permeabilizing activity against a broad spectrum of fungi and bacteria. These activities have propelled omiganan into clinical trials [31]. In one study, the antifungal activity of cathelicidins belonging to three distinct structural classes including: α- helical SMAP-29, BMAP-27, BMAP-28, β-hairpin—PG-1 and linear—indolicidin was reported [47]. These substances showed rapid, concentration-dependent, antifungal activity against most of the examined yeast strains and some filamentous fungi (*C*. *albicans*, *C*. *neoformans*, *Rhodorotula*, *Pichia*, *Aspergillus* spp. and *Penicillium* spp.) irrespective of their resistance to classical antimycotics [47]. Similar to LL-37, ceragenins display strong activity against a variety of bacteria [24–26]. However, as indicated by our current data, ceragenins are effective at much lower concentrations against yeast and filamentous fungi than LL-37, and the antifungal activities of CSA-13, CSA-131 and CSA-192 vary depending on the fungal strain. Generally, cathelicidin LL-37 exerts some ability to inhibit the growth of the *Candida* strains tested but only at relatively high concentrations. The MICs/MBCs values for LL-37 are 10–100 higher than those reported for LL-37 concentrations at the mucosal surface or in human body fluids [48]. However, careful analysis of our results reveals that data from killing assays and MIC/MBC evaluations are not always compatible with each other. We suggest that these variances results from nature of experimental settings, considering the differences in employed environment (PBS/high nutrient medium) and varied duration of experiments [49].

In our experiments, the MICs/MBCs values for ceragenins against tested species are comparable to amphotericin B (AmB). According to breakpoints indicating by the Clinical and Laboratory Standards Institute (CLSI) and by the European Committee on Antimicrobial Susceptibility Testing (EUCAST), all four tested *Candida* strains are resistant to fluconazole and sensitive to AmB [50]. In general, fluconazole is still found to be active against most isolates of *Candida*, but in institutions with high proportions of *C*. *glabrata* and *C*. *crusei*, elevated rates of resistance to fluconazole and newer azoles (voriconazole and posaconazole) are observed. These situations drive the use of echinocandins as first-line therapy for moderate to severely ill patients [51]. The observed low AmB MICs are in agreement with the results of a study by Montagna et al. [52], who reported that this antifungal is highly active against many clinically relevant yeasts. Although AmB has been considered the gold standard for many decades in the treatment of invasive fungal infections, acquired resistance is still rare. Unfortunately, clinical use of AmB is limited by its poor aqueous solubility, toxicity and infusion-related reactions (mainly fever and chills) [52].

Our results demonstrated that all tested ceragenins possess higher fungicidal activity than omiganan, which is being developed as a topical gel for prevention of catheter-associated infections. The fungicidal activity of omiganan against a broad spectrum of *Candida* species and molds (both *Aspergillus* spp. and non-*Aspergillus* spp. fungi) followed by lack of detected resistance to this peptide-based agent, resulted in entry of this compound to the clinical trials [53]. However, in our experimental settings, omiganan in doses ranging from 1 to 100 μg/mL was ineffective in eradicating tested *Candida* strains. MIC/MBC values obtained in our study were consistent with the results presented by Fritsche *et al*. indicating the range of MIC values from 32 to 256 μg/mL (18.0 μM-144.0 μM) for *C*. *albicans* isolates [53]. Importantly, these values are substantially higher than those seen for ceragenins (ranging from 0.6 to 36.5 μM).

The mechanism of action of ceragenins against fungi is not precisely defined but as derivatives of cholic acid that mimic the morphology of natural AMPs, they are expected act in a similar manner, causing damage and dysfunction of the plasma membrane. Our experiments with fluorescently labeled LL-37, CSA- 13 and CSA-131 support this idea; all compounds quickly localize to the *C*. *albicans* cell membrane. Additionally, images taken using SEM and AFM show that both compounds target plasma membranes as indicated by deformed cell surfaces and extensive changes in *Candida* cell shape. It is possible that the precise mechanism of action of ceragenins differs from LL-37, since ceragenins antifungal potential is superior. This hypothesis is supported by reports indicating differences in fungal cell morphology and biophysical properties after treatment with agents characterized by different mechanisms of action [39]. However, differences may also reflect the susceptibility of LL-37 to proteolytical degradation. The antifungal action of human LL-37 and another cathelicidin, chicken CATH-2, have been described by Ordonez *et al*. [54]. They show that LL-37 eradicates *C*. *albicans* very rapidly and independent of the energy status of the fungal cell in a process that involves cell membrane permeabilization and simultaneous vacuolar expansion. The authors suggest that the membrane destabilization effect is the most important mechanism of cathelicidin antifungal action. On the other hand, other internal targets may also be involved. Interestingly, in the same report, comparison with another antifungal peptide, histatin 5 (Hst5), shows that although MFCs are similar for the three examined peptides, the mechanisms of antifungal activity slightly differ. Hst5 activity is energy-dependent and is mainly directed to the fungal vacuole [54]. Similar to activity against bacteria, membranes are the main targets associated with LL-37 antifungal activity. On the other hand significant differences in the cell surface of the *C*. *albicans* and bacteria require much more detailed studies to justify the above statement. The fungal membranes are less negatively charged compared to bacteria as they contain neutral sterols [55]. Additionally, yeast cell walls are thicker and composed of different polysaccharides such as chitin and ß-glucan. It is also important to note the differences between mammalian and fungal membranes, since these might explain the selectivity and lower ceragenin toxicity towards human cells.

Because most recurrent mucosal candidiasis infections as well as fatal fungal invasive infections result from pathogenic biofilms, we aimed to assess the influence of LL-37, CSA-13 and CSA-131 on the *Candida* biofilm formations in presence of DNA, which is a well-defined factor stimulating biofilm formation and playing an important role in *Candida* protection from the host immune system [13]. Our results confirmed the stimulatory effect of DNA on *Candida* biofilm formation. Although all three tested compounds significantly inhibited the DNA-dependent development of early biofilms, some differences were visible during mature biofilm formation, where ceragenin activities were much stronger than those of LL-37, which did not maintain its inhibitory effect when biofilm growth was continued over 24 h. It might be speculated that LL-37 acts mainly in the early stages of biofilm formation in contrast to ceragenins, which seem to be active during the early and prolonged periods. Perhaps, during the early stages of biofilm development the amount of available eDNA to which cationic antimicrobials can bind is small, but after 48 h as the biofilm matures, the amounts of eDNA increases and the interaction between negatively charged eDNA and cationic antimicrobials is more likely to occur, reaching levels high enough to inhibit LL-37. On the other hand, the stability of ceragenins due to lack of susceptibility to proteolysis may contribute to the higher activity than LL-37. Among classical, commercially available antifungals, echinocandins display *in vitro* activity against mature *Candida* albicans biofilms but were not effective in patients with *Candida* device-associated biofilm infections. According to various guidelines, effective treatment includes device removal in addition to treatment with antifungals [56]. The antibiofilm activity of ceragenins represents a promising option for the future development of antifungal agents. During *Candida* biofilm formation, eDNA seems to assure biofilm maintenance and stability and may be species/strain dependent [57]. Our data are in agreement with previous reports that enzymatic destruction of eDNA decreases biofilm biomass and leads to enhanced activity of antibacterial agents [15]. DNase I, when used in combination with LL-37 or ceragenins, inhibits biofilm formation better than DNase I alone. The ability of DNase I to restore LL-37 and ceragenin activity against biofilm, as shown in our experiments, is likely associated with the interaction of antibacterial compounds with DNA, giving complexes which diffuse poorly through the biofilm matrix. DNase I breaks up these complexes and liberates the antimicrobials. Similar observations using bacterial biofilms formed by *Haemophilus influenzae* in chronic otitis media infections were previously described [58]. Indeed eDNA interacts with human β-defensin-3 (hBD-3) which is critical for protection of the middle ear and diminishes hBD-3 biological activity. Formation of *Haemophilus influenzae* biofilm in the presence of DNase I /recombinant hBD-3 resulted in a significant reduction in the biofilm mass restoring the antimicrobial activity of the hBD-3 [58]. DNase ability to inhibit biofilm development might be further explored to develop new treatment strategies of various chronic diseases associated with biofilm formation [59] such as chronic lung infection in cystic fibrosis patients or chronic otitis media [58]. Moreover, DNase might be used to increase antimicrobial activity of new potential antibacterial and antifungal drugs in treatment infections associated with biofilm. In agreement with previous reports showing the advantage of ceragenin antibacterial activity over LL-37 in experiments with bacterial pathogens [60], we have observed a similar pattern comparing LL-37 antifungal activity to that of CSA-13 and CSA-131. Moreover, some other limitations of cathelicidin LL-37 activity at infection sites were previously reported, including a significant decrease of LL-37 activity in blood plasma, pus, saliva and urine [48, 61]. Various factors present in human body fluids, such as proteases, ion concentrations, pH, the presence of apolipoprotein A in serum, glycosaminoglycan in wound fluid and mucins in saliva can influence the antimicrobial activity of LL-37 [62, 63]. Our observations show strong candidacidal activity of ceragenins in the presence of body fluid, which is in agreement with previous reports documenting that the non-peptide antimicrobial molecules are more resistant to many of factors that inhibit antimicrobial activity of peptides such as proteases [61]. Indeed the compounds present in saliva, urine and pus did not alter the candidacidal activity of CSA-131 and only partly limited CSA-13 activity (Fig 8). In our experimental setting, both ceragenins retained more than 50% of their activity in plasma. It is possible that higher doses of these agents may be sufficient for effective eradication of *Candida* from infection sites and that the increased dosage is possible due to previously reported their good safety profile [64].

## Conclusion and Perspective for the Future

The experiments described here demonstrate that ceragenins CSA-13, CSA-131 and CSA-192 have stronger candidicidal activity than natural LL-37 peptide and omiganan against all tested fluconazole-resistant yeast cells as well as against young and mature biofilms. Moreover the differences between CSA-13 and CSA-131 indicate that molecular structure modifications can alter antimicrobial activity and their resistance to inhibitory actions of some body fluids. The activity of ceragenins in body fluids and membrane-dependent, relatively unspecific mode of action show a promising opportunity for the development of new antifungal strategies. Additionally, we confirmed that ceragenins possess high fungicidal activity against a broad spectrum of pathogenic fungi. Further studies, especially involving animal models should be conducted to assess ceragenin potential use as antifungal drugs in systemic and in topical applications either alone or in combinations with DNase I.
